# Supplementary material for: A learning-based method for online adjustment of C-arm Cone-beam CT source trajectories for artifact avoidance
Source: Int J Comput Assist Radiol Surg. 2020 Aug 25;15(11):1787–96. doi: 10.1007/s11548-020-02249-1 (PMC7603453; doi:10.1007/s11548-020-02249-1)
Supplement: Supplementary file 1 — Supplementary material 1 (pdf 347 KB) [file 11548_2020_2249_MOESM1_ESM.pdf]

Noname manuscript No.
(will be inserted by the editor)

Supplementary material

Mareike Thies[1,2,*] · Jan-Nico Zäch[3,*] · Cong Gao[1] · Russell Taylor[1] · Nassir Navab[1] · Andreas Maier[2] · Mathias Unberath[1]

Received: date / Accepted: date

E-mail: unberath@jhu.edu
[1] Laboratory for Computational Sensing + Robotics, Johns Hopkins University, Baltimore, MD
[3] Pattern Recognition Lab, Friedrich-Alexander-Universität Erlangen-Nürnberg
[2] Computer Vision Laboratory, Eidgenössische Technische Hochschule Zürich
[*] Both authors contributed equally and are listed in alphabetical order.

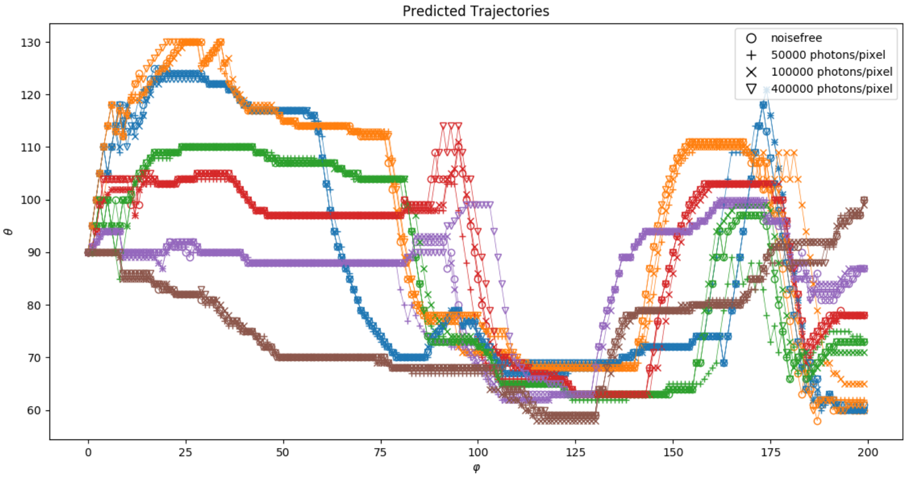

Fig. 1 Trajectories predicted for different noise levels of the X-ray projections. All acquisitions are plotted in one color for each screw-pair and start at the same out-of-plane angle of 90°. The different noise levels are indicated by different markers: Circles correspond to the noisefree acquisitions, crosses to $5 \cdot 10^4$ photons per pixel, x to $1 \cdot 10^5$ photons per pixel and triangles to $4 \cdot 10^5$ photons per pixel.

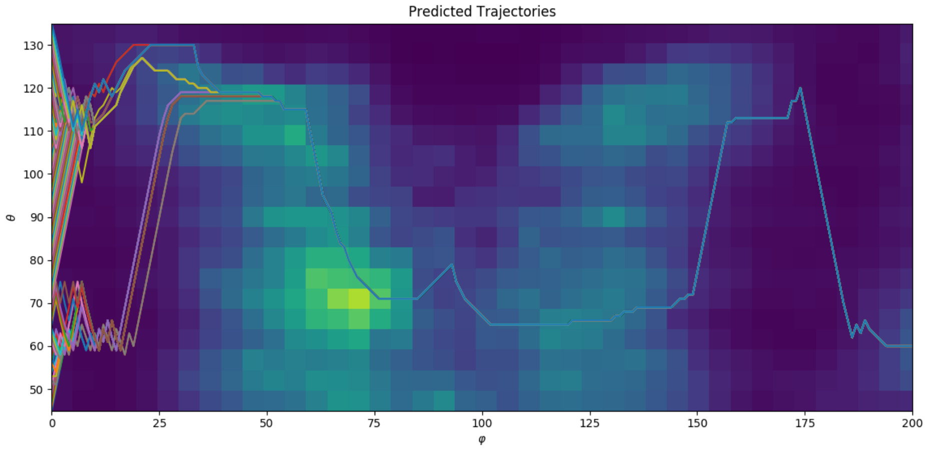

Fig. 2 Trajectories predicted for all out-of-plane starting angles in the range $45° \leq \theta \leq 135°$, with an increment of $1°$. The background image corresponds to the groundtruth detectabilities and the plots indicate the chosen trajectories.
